# Narratives of health-promoting experiences by older husbands and wives providing care to their home-dwelling spouses receiving home-care services in Norway

**DOI:** 10.1186/s12913-024-12097-3

**Published:** 2024-12-18

**Authors:** Hege Stokmo Melilla, Beate Lie Sverre, Grethe Eilertsen, Siri Tønnessen

**Affiliations:** 1 Department of Nursing and Health Sciences, Faculty of Health and Social Sciences, University of South-Eastern Norway, 215 Raveien, Borre, Vestfold 3184 Norway; 2 Department of Nursing and Health Sciences, Faculty of Health and Social Sciences, University of South-Eastern Norway, 58 Grønland, Drammen, 3045 Norway; 3 USN Research Group of Older Peoples’ Health, University of South-Eastern Norway, 58 Grønland, Drammen, 3045 Norway; 4 Lovisenberg Diaconal University College, 15B Lovisenberggata, Oslo, 0456 Norway

**Keywords:** Health promotion, Older family caregivers, Home-care services, Home-dwelling spouses, Narrative design, Qualitative method

## Abstract

**Background:**

In today’s healthcare systems, older family caregivers who care for their spouses at home are indispensable providers of healthcare. However, many of these caregivers are at risk of becoming ill themselves. To prevent this and to guide the development of targeted healthcare services, home-care personnel need knowledge on how to promote the health of older family caregivers. The purpose of this study was to understand the health-promoting experiences of older family caregivers who care for their home-dwelling spouses receiving home-care services.

**Methods:**

The experiences of older family caregivers were explored using a narrative design that involved narrative interviews and a narrative thematic and structural analytical approach. The sample consisted of four husbands and six wives aged 79–91 years. In the analysis, two storylines of narratives were constructed: one by husbands and one by wives.

**Findings:**

In the husbands’ narrative, *continuation of everyday life* and *social support in everyday life* were highlighted as being health-promoting. In the wives’ narrative, the importance of having *time for oneself* and *being seen, heard and included by the home-care personnel* were emphasized as promoting their health. The husbands’ narrative contained stories of the past and the present, while the wives’ narrative mainly comprised stories of the present.

**Conclusion:**

This study has provided insights into the narratives of older husbands and wives acting as family caregivers regarding what promotes their health caring for their spouses at home. Their stories show variations in care style, coping style and experiences of the caregiver burden. These findings suggest that home-care services should consider providing customized health-promoting services to older family caregivers who are providing care to their home-dwelling spouses receiving home-care services. Given the sample size of only ten participants, further qualitative and quantitative research is needed.

**Supplementary Information:**

The online version contains supplementary material available at 10.1186/s12913-024-12097-3.

## Introduction

Family caregivers are indispensable providers of long-term informal care globally [1, 2]. Across country members of the Organization of Economic Cooperation and Development (OECD), about 13% of those older than 50 years provide informal care on a daily or weekly basis, and three out of five of the daily carers are women [3]. In Norway, approximately 110,000 people older than 67 years receive home-care services [4]. Most of them have multiple illnesses, averaging four or five conditions [5], and 40% of those older than 70 years who receive home-care services have dementia. Other common conditions include age-related physical ailments such as heart disease, diabetes, chronic lung disease, mental illness and cancer [6, 7]. These conditions require different types of assistance, including personal care, housekeeping, mobility, food preparation, emotional support, financial support and medical care [8]. Aging at home is preferred by most older adults and supports government policies, since home-care services are generally more cost-effective [9, 10]. The increasing number of older people in need of care along with the concomitant shift towards home-based care are increasing the effort required from both informal and formal caregivers [1, 11, 12].

Providing daily care can be rewarding for family caregivers, but also demanding and carries the risk of them becoming ill themselves [13, 14]. This is particularly evident for older family caregivers providing spousal care given that advancing age often leads to health decline and loss of function [15]. The ability to age healthy can vary markedly depending on factors such as age, culture and gender [16]. Furthermore, the home environment commonly has a positive impact on the health of older family caregivers, by serving as a connection to the past and as an arena for social interactions with family, friends and the neighbourhood. The importance of the home as a setting for everyday life increases with age as people spend more time there [17]. When home-care services are integrated into the home setting, they become part of the everyday life of the family caregiver. Moreover, home-care personnel can provide caregivers with relevant support when they have access to the experiences of these caregivers. Steps have been taken in OECD countries to promote such support for family caregivers from home-care personnel, including information, counselling, training and respite. However, this support is generally still inadequate [3].

In most Nordic countries, the municipalities and their home-care services are legislated to provide support to family caregivers who have long-term, extensive responsibilities [3, 18]. In Norway, legislated support provided to caregivers includes respite services such as day-care centres and short-term stays in institutions, as well as training and guidance to help caregivers manage their caregiving tasks. Additionally, a caregiving allowance is available for those performing heavy caregiving tasks that would otherwise be available from municipalities [19]. Municipalities are obliged to offer psychosocial support, including access to counselling and support groups [8]. However, the extent to which this is systematically followed up is uncertain. Home-care services in Norway are provided free of charge, funded by the Norwegian welfare state [18], which ensures that all residents have a universal right to receive the healthcare services that they need [20]. Delivering customized and sufficient support requires the home-care personnel to understand what is experienced as health-promoting by the older family caregivers. Strong support of the health of family caregivers by home-care services means that they can provide better care to their home-dwelling spouses [21].

### Background

Research on family caregivers has been conducted since the 1980s. Early studies of the impact of caregiving revealed widespread experiences of caregiver burden by family caregivers [22–25]. The concept of *caregiver burden* encompasses the physical, psychological, emotional, social and financial stresses that they might experience while providing care [26].

A more recent scoping review indicated how being a family caregiver in old age can be burdensome, increase psychological distress and decrease the health-related quality of life [27]. Additionally, previous research has revealed interrelationships between the care burden experienced and the health and frailty of the caregiver, and demonstrated that health failures can lead to care breakdown [28]. The burden can be increased further by sleep disruption [29]. A deteriorating health condition of the care-recipient will increase the required caregiving tasks, including assistance in performing the activities of daily living, and hence impact the care burden experienced by the caregiver so as to impair their cognitive and physical functioning [23, 30]. Moreover, the quality of the relationship between the caregiver and care-recipient influences the experienced care burden, with a good-quality relationship decreasing the burden [31]. From a cultural perspective, women rather than men are traditionally expected to take on caregiving roles [32], and female caregivers might receive less social support than male caregivers, which may increase their perceived burden [33]. A Norwegian study found that male caregivers received 19% more public assistance than did female caregivers, which can decrease their perceived burden [34]. High-quality social support from close family and friends and municipality caregivers is related to a lower perceived burden among both female and male caregivers [23, 35], although male caregivers tend to be more reluctant to seek such support [36].

Some former studies have also identified gender differences in the caregiver burden, with older wives reporting that they experience higher levels of burden [37], depression and distress [38] than older husbands, while other studies found no gender differences in burden [39]. The systematic review of Zygouri et al. [40] that included 70% older caregiving spouses revealed gender differences related to care and coping styles. While older husbands commonly used a task-oriented, managerial approach that often decreased their care burden, older wives tended to apply an emotional and relational approach that frequently increased their care burden. The heavier care burden among wives was connected to subjective factors such as relational and financial problems, and prioritization of daily activities and caregiving tasks as noted by Swinkels et al. [33]. Unlike those two previous studies, the recent quantitative study of Pacheco et al. [41] that measured gender differences in family caregiving found that women spent more time providing care but were as satisfied as their husbands with their health status.

The term “caregiver burden” has been critiqued due to its potential to undermine the strengths, resources and positive aspects of caregiving [24]. This prompted the initiation of studies exploring positive aspects of caregiving during the late 1980s [42]. Recent studies have highlighted the satisfying aspects of caregiving to spouses with dementia among older family caregivers. Satisfying aspects are related to the appreciation of relationships, comfort and togetherness, increase in spiritual well-being, finding meaning in the disease, extra time spent with spouse and family, improvements in relationships, and increased meaning and purpose in one’s life [43]. Moreover, mindfulness, which refers to the ability to live in the moment, is linked to positive outcomes of older family caregivers [44]. A quantitative integrative review examining health-promoting behaviours adopted by adult children and older spouses as family caregivers, found that the search for information, family counselling support, and social, emotional and spiritual support were important to promote quality of life and well-being [45].

Despite the growing body of knowledge on the positive aspects of being an older family caregiver, much of the previous research has been primarily quantitative, related to a specific diagnosis of the spouse, included participants of all ages or has not specified the home as a setting. Therefore, there is a need for qualitative research to understand how to promote the health of older family caregivers during their daily efforts. Hence, the aim of this study was to provide insights into the health-promoting experiences of older family caregivers who care for their home-dwelling spouses receiving home-care services. This knowledge is essential to enable home-care services to deliver individualized and targeted healthcare, both to the older family caregivers and consequently to their home-dwelling spouses.

### Theoretical framework

The present study was based on the salutogenic theory on health promotion of Antonovsky [46]. Salutogenesis refers to the origin of health while emphasizing factors that promote health rather than disease. This theory posits health as a continuum between ease and dis-ease, which challenges the dichotomous view of health as the state of being either sick or well and promotes a resource- and strength-based perspective on health. Central to this theory is the concept of “sense of coherence” (SOC), which is a stable attitude that determines how stressors are perceived as comprehensible, manageable and meaningful; though, radical and long-term changes in a person’s life situation can alter this. However, subsequent developments of the theory have suggested that SOC is a continuous process rather than being stable [47], implying that it may develop during the lifespan and so can improve with age [48]. Antonovsky [46] considered that the following general resistance resources can strengthen the SOC: social support, ego strength, financial status and cultural setting, as well as the ability to flexibly use these resources. Research has indicated that a strong SOC can help to protect older family caregivers from the burden of caregiving and even promote their health [49].

## Method

### Narrative design

An explorative narrative design [50, 51] was chosen to explore older family caregivers’ experiences of what promotes their health when caring for their spouses at home. In this study a story was treated as a detailed account, and valued as a construction and *mimesis* [50] or retelling of the actual situation in the way it was remembered by the caregiver. Essential to this process is how the participants construct meaning and their identity through discourse and communication, and thereby constantly creating themselves [51]. Information about the context is central to understanding the experiences of the older family caregivers, and is embedded in data construction, analysis and writing [51]. Furthermore, the *story* was treated as accounts of what happened as told by the participant, while the resulting *narrative* was considered to be the outcome of the analyses performed by the researchers [52].

### Participants, recruitment and final sample

A purposeful sampling method was chosen to recruit participants who could provide nuanced and rich data representing both gender and socioeconomic backgrounds [53] from diverse central and rural areas of a municipality in the eastern part of Norway. The following inclusion criteria were applied: spouse aged ≥ 75 years, family caregiver living with a home-dwelling spouse in need of care, spouse receiving help from home-care services regularly, and minimum score of 2.2 in the International Classification of Functioning, Disability and Health (ICF), which indicates an extensive need for personal assistance [54]. An ICF classification of 2.2 was used to ensure that the care-recipients had significant care needs that were likely to require their caregivers to perform considerable caregiving tasks.

Participants were recruited in collaboration with a municipal team who were familiar with the ICF framework and had access to home-care services. One of the 11 caregivers who were invited to participate declined, and so the sample comprised ten participants. The home-care personnel brought the information sheet describing the study during a home-care visit and returned the signed consent form in a sealed envelope to the municipal team. The envelope was then handed to the first author, who established contact by phone with the older family caregivers and arranged dates and times for individual interviews with the ten family caregivers. The final sample comprised six women and four men ranging in age from 79 to 91 years and residing in different areas of the municipality (Table 1). The conditions of the care-recipients were largely consistent with the national statistics described in the Introduction, with the exception of having more cognitive impairment. All had multiple diagnoses, were frail and needed extensive care.

**Table 1:** Characteristics of participants, housing types and home-care services of care-recipients

Name^a^	Age, years	Years married	Housing type	Home-care services	Respite care
Jack	87	34	Apartment	Twice daily	None
Steven	80	55	Detached house	Four times daily	Day centre once weekly
Liam	83	63	Detached house	Twice daily	None
John	79	56	Terraced house	Once daily	None
Lily	75	54	Detached house	Five times daily	Day centre five times weekly Flexible respite 70 days annually
Ann	75	15	Detached house	Three times daily	Husband refused day centre and respite care
Clara	72	50	Apartment	Four times daily	Day centre five times weekly Flexible respite 70 days annually, but husband refused respite care
Carly	80	56	Detached house	Three times weekly	None
Lynn	87	66	Apartment	Once daily	Husband refused respite care
Barbara	85	57	Apartment	Twice daily	None

^a^Fictious names

### Setting

The interviews took place in the participants’ homes, which was their stated preference. To be in familiar surroundings is considered to enhance the feeling of safety and to build trust and confidence in revealing inner thoughts [55]. Thus, the context of home most likely influenced how the stories were told by the participants and was therefore central to the assessments performed [56]. The contextual information revealed the interest that the participant had in literature, art and gardening, as well as the maintenance level, orderliness and layout of their home. This information expanded the first author’s understanding of the participants, and was used as conversation topics to show recognition and to promote a relational connection [51].

### Data construction by narrative interviews

To promote the provision of long and detailed stories, the narrative interviews were conducted as conversations between two active participants that involved turn-taking as well as entrance and exit talking. The unstructured interview guide contained one overarching open-ended question supplemented by four subquestions that were woven into the conversation, in line with Riessman [50] (Table 2). An extended version of the interview guide is available (Additional File 1).

**Table 2:** Interview guide

Please tell me how you have experienced being the wife/husband of … (name of husband/wife) after he/she became ill
Additional subquestions:
• Can you tell me about an enjoyable moment you have experienced as a caregiver?
• Can you tell me about a difficult moment you have faced as a caregiver?
• How have your interactions with home-care services been?
• What could improve your current life situation?

To enhance relevance and the health-promoting perspective, the questions were developed in collaboration with representatives of family caregivers and employees in the healthcare services.

The first author conducted the interviews. To promote storytelling, the first author tailored the interview questions to fit the descriptions of the life journeys of the participants, which facilitated the sharing of their experiences. To ensure richness, the older family caregivers were asked to elaborate by asking the following questions: “Can you please tell me more about that?” and “How did you feel when that happened?” Following the recommendations of Riessman [51], the first author aimed to listen attentively in an emotionally engaged way. The interviews lasted 55–85 min and were digitally recorded and then transcribed verbatim by the first author. Notes were made after each interview on the immediate impression of the story and its local context.

### Ethical considerations before, during and after the interviews

Approval for the study was granted by the Norwegian Agency for Shared Services in Education and Research (June 29th, 2021). Written informed consents were obtained from the older family caregivers. The aspects of voluntary participation and the option to withdraw from the study at any time without consequences were emphasized both in writing and orally. The data were managed in line with guidelines for confidentiality to protect the participants’ identity. Deidentification was ensured during transcription and when subsequently presenting the findings. All participants were given fictious names.

Audio files and their transcripts were kept separately from the contact information in secure locations. Reflections regarding the first author’s preconceptions and background were considered since they affected the interviews and the interpretation of the obtained data [55]. For example, being an insider of the field as a female mental-health nurse who had previously worked in the home-care services provided a special awareness of interactions and knowledge of a person-centred ethical approach. This manifested as politeness, thoughtfulness and being attentive to nuances to create a safe atmosphere and to establish trust so that the family caregivers could open up during the interviews. Finally, the participants were asked how they experienced the interviews. These ethical precautions were taken to increase the validity and trustworthiness of the obtained data [51].

### Narrative thematic and structural analyses

The ten interviews formed the basis of systematic narrative, thematic and structural analyses [50, 51]. The narrative analysis helped to interpret the meaning of the stories as a whole, the thematic analysis allowed searching of patterns across stories, and the structural analysis illuminated how the stories were organized in terms of the use of past, present and future tenses [51]. The analyses were performed collaboratively among all of the authors. The first and last author read and analysed all of the interviews, while the other two authors read and analysed two interviews each. All of the authors met several times during the process to discuss and reach consensus on the tentative findings.

The authors initially read the transcribed interviews separately several times to gain an overall understanding [51]. The preliminary impression was that all of the older family caregivers told stories about how their lives had changed after their spouses became ill, emphasizing how they managed these changes. The interviews were then read and coded individually, followed by a search for common patterns across the interviews. This process was guided by the overall aim of the study: to understand the health-promoting experiences of the family caregivers. This procedure resulted in the construction of ten subthemes. These subthemes suggested that health-promoting aspects were perceived differently by husbands and wives, with four subthemes reflecting the husbands’ experiences and six subthemes reflecting those of the wives. This insight led to the construction of four themes organized into two narratives: one for the husbands and one for the wives. The husbands’ narrative included the themes of *continuation of everyday life* and *social support in everyday life*, whereas the wives’ narrative comprised the themes of *time for oneself* and* being seen, heard and included by the home-care personnel.*

Finally, excerpts from the interviews were selected to reflect the ten subthemes. Each subtheme was illustrated by an excerpt from one interview, chosen for its richness and detail, and subsequently written as a typology.

The structural analysis, where the temporal perspective constituted the structure, revealed that the family caregivers emphasized the past and the present differently, with the husbands’ narratives connected both the past and present contexts, while the wives’ narratives in general unfolded in the present contexts.

**Fig. 1:**
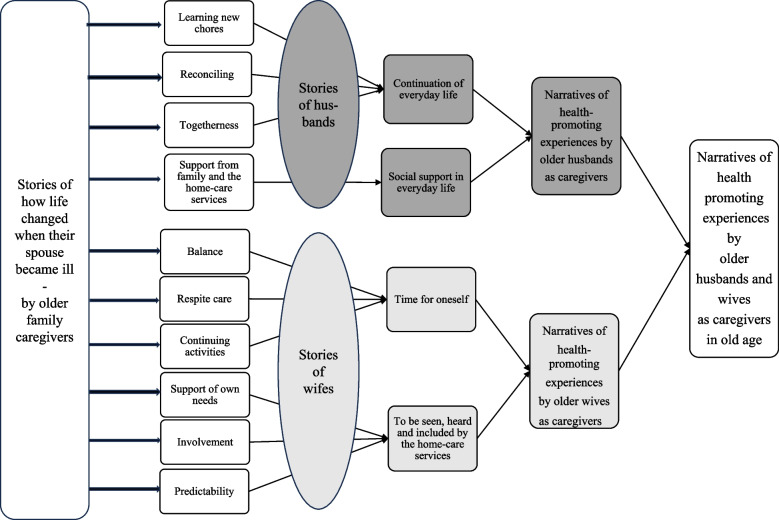
Development of subthemes, themes, narratives and main plot

Lastly, the main plot was interpreted as older husbands and wives undergoing major life changes when their spouse became ill. However, the experiences of the husbands and wives differed in terms of what aspects promoted their health. The husbands made connections between the past and the present to find meaning in their current situation as carers, while wives tend to need time for themselves and struggled in the present with a loss of self and a lack of support as carers (Fig. 1).

## Results

The results of the analyses of the health-promoting narratives of older family caregivers caring for their spouses at home receiving home-care services are first presented by considering the main plot. Thereafter, the two narratives of the participants health-promoting experiences are presented, including the interpretation of the four themes and the subthemes.

### Health-promoting experiences in old age

The husbands and wives underwent major life changes when their spouses became ill, implying a relational shift in old age with life-changing experiences of health promotion when becoming a family caregiver to their spouse. The husbands made connections between the past and the present by continuously solving tasks to find meaning in their current situation as carers, while wives tended to need time by their own, but in the absence of this struggled with a loss of self and a lack of support as carers. The experiences of the husbands and wives in terms of what they considered health-promoting in this situation is elaborated below in two narratives.

### Narrative of health-promoting experiences of older husbands as caregivers

The husbands connected the past and the present by conducting retrospective memory recalls and elaborating positive experiences, holidays and happy times. Traditions and common family activities had been important to them and were still a part of their present life and identity. They highlighted the importance of the *continuation of their everyday lives* and *receiving social support* when encountering their life-changing situation.

### Continuation of everyday life

All of the husbands recognized how their lives had changed and provided considerable details about their new tasks and responsibility. They were used to a gender division of chores, but now they had taken on household chores such as cooking, cleaning, planning meals and purchasing food in addition to the responsibilities they had previously. They highlighted the importance of holding on to everyday routines and traditions and developed new skills to cope with and control the changed situation. They demonstrated a positive attitude towards their obligation as carers. By coping with the new demands, the normal activities of everyday living could continue. The older husbands seemed to have found a balance regarding their changed situation. The story of Liam about *learning new chores* illustrated this:

*You know, when the kids grew up, I was away for years, so my wife was responsible for the child-rearing and the household chores. I have usually taken care of the heavy work; I was a handyman from an early age. But after she became ill, I have had to take on some extra chores. To say it simply, everything she did before I do now. I do it because I like to hold on to things, I am used to that. I am very bound by traditions and routines. I am conservative and like conventions you know. I am doing fine, but there is a limit for me as well, but I have not reached that limit yet.*

The husbands seemed to have accepted, reconciled and found meaning with their changed situation. Some of them talked about previous hobbies and interests that were important to them but which they had to stop participating in. They gave the impression that they accepted this change and had found other activities to do at home. It appeared that they understood that life naturally changed in old age and that they searched actively for solutions to support the continuation of their everyday lives. Their motivation to change their situation was additionally linked to a sense of loyalty to their wives. John gave this example on *reconciling*:

*We used to be highly active, but when Liv became ill everything changed. She had problems with her legs for years, but in the beginning, we still travelled to Europe with a walker and wheelchair. We had to quit travelling due to complications after her stroke, so now we just stay here at home. We enjoy meals, listen to music, watch television and collect stamps together. I am never bored, I have a workshop in the garage where I make small furniture, and in summer I grow vegetables. Things usually work out. So, you know I am fine with this situation if Liv is content. That is the most important thing to me, and that my health lasts*.

Several of the husbands gave the impression that the quality of their own lives was strongly associated with how their wives experienced the situation. Some of the husbands referred to the marriage wows as being important, and a few of them recounted that they had promised to look after each other “in good and bad times”. Their friendship and common interests were highlighted. They seemed overly attached to their wife and wanted to share time together. Some of the husbands stated that they felt lonely if their wife was not present at home. When their wife was present, everyday life continued, and they could be together as usual. Jack’s story about *togetherness* demonstrated this:

*A year ago, Laura fell and broke her back and was admitted to the hospital. She had to undergo training for weeks. That period was not pleasant. You know, the house was completely empty, so silent and quiet. Laura was up there not feeling well, and I was here not feeling well. It would have been better if she were at home. It was as if one of the walls were missing then*.

Continuation of the everyday lives with their wives made sense to the husbands. They adapted to new challenges and learned new tasks. When the scope of tasks became too great, they happily welcomed help from home-care services.

### Social support in everyday life

All of the husbands talked about the turning point when they were unable to cope with all the care needs of their wives and the home-care personnel visited their homes on a regular basis. The home-care personnel helped with hygiene and medication needs and arranged for housing aids. In general, the husbands were grateful to receive this extra support, and overall they felt content with their situation. Most of them experienced that the home-care personnel could understand their needs. For some husbands, their family was also important when it came to providing practical and mental support and guidance. Liam’s story gave an example of the value of *support from family and home-care services*:

*It all started 5 years ago, she began to forget things, and everything grew out of hand. At one point I could not manage by myself and then I applied for home-care services. When they came everything changed rapidly. First, we got an emergency alarm and a fire alarm, she got a support for her bed, a tempura mattress and adjustable bed base. They removed the carpets to prevent her from falling. They saw what was necessary, and I was impressed with the system. They started to come twice a day, and this frequency was sufficient. Also, my son is of great support. He is a professional and has knowledge about her illness. I could not manage without him either. So, it is fine. The only thing that bothers me is that you never know when the home-care personnel will show up. Like today I had to call them at 11:15 and ask if they totally forgot about her, so that is difficult for me*.

Overall, the husbands were content with their situation and welcomed the home-care personnel into their homes and were also grateful for the support from their family. They did not go into detail about how this support made them feel mentally, but it seemed important that the home-care personnel were professionally trained, acted calmly and provided necessary information in acute situations.

### Narrative of health-promoting experiences of older wives as caregivers

The stories of the wives generally took place in the present time and described their current situation as difficult. The wives highlighted that essential elements contributing to their well-being were having time for oneself and being seen, heard and included during the visits of the home-care personnel.

### Time for oneself

The wives also told stories about how their husband’s illness had turned their lives upside down, and many found the changed situation challenging. To be able to cope with the situation and to recover, to have time for oneself was important. They spent this time participating in different activities such as exercising, hiking or listening to lectures, or with family or friends. They elaborated how their husband’s illness had developed and how this had affected them both mentally and practically. Ann’s story about *balance* demonstrated this:

*I only noticed it about five years ago because he started forgetting. You know, he has changed a lot over the last few years, he has become so angry and self-absorbed. He decides everything that happens in the house. So, my life is completely gone. I cannot invite guests, listen to the radio or watch television. All of this makes me feel like I have no value. So, some hours of alone time outside the house and activity are my salvation. That is it. That activity includes both dancing and aerobics. Then I talk to others a little bit, I can be myself and that gives me the strength that I need. I need these moments so that I get physically and mentally back into balance*.

The wives who received adequate respite care were more satisfied with their lives despite the heavy care burden because they then had time for oneself to perform the domestic household chores and other activities meaningful to them. Lily shared this experience regarding *respite care*:

*In the beginning I was very worried since the disease was sneaky. But now it is fine because his situation is stable and it works out with home-care services, respite care and relievers. These services are important because he must have round-the-clock follow-up. I can be a little social, go to the library, listen to lectures or visit our cabin. So, I am doing well*.

In some cases, the husbands had considerable care needs, and consequently their wives were not able to have time on their own either inside or outside the house. They felt isolated and lonely, overwhelmed by the huge responsibility. This had dramatic consequences for how they experienced their situation, and some felt like they were losing themselves. The importance of *continuing activities* was emphasized. Clara’s story exemplified this:

*During the last two and a half years there has been a huge deterioration, and you know, it totally changes your life. Everything is on me now. So, you become unsociable, locked in, you are not free in your own home, you are kind of resigned, you are on hold. Before, I spent a lot of time outside, went for walks, was in a choir, was social. Now I am struggling to get to the hairdresser. My arms are exhausted from the lifting, so I went to physical therapy, but I just had to stop going because I could not make it there on time. So, everything is complicated now. My own life activities are now restricted to going for a walk on the terrace, mowing some lawn. My house has become an institution, and I ask myself, am I a wife or a personal assistant? I have lost myself*.

The immense responsibility made life difficult for some of the wives who had to give up their personal interests, and in some cases, they could not receive necessary medical treatment. They needed time for oneself but had difficulties leaving the house since their husband could not stay alone. They needed support from home-care services.

### To be seen, heard and included by the home-care personnel

Central to the wives’ stories were how home-care services affected their lives. Most of them highlighted the importance of receiving these services. For many, their husband’s illness progressed over time, and eventually the total burden became too great, which led them to apply for home-care services. Several of the wives said they were exhausted and needed their husband to go into respite care so that they could recover. But when their husband refused, this was immediately respected by the home-care personnel regardless of whether or not the husband had a diagnosis of dementia. The husbands’ wishes to stay at home were given priority over the wives’ need for respite. The wives wanted to be seen and heard by the home-care personnel and needed support, including in encouraging their husbands to accept respite care when this was necessary. Ann’s story about *support of own needs* illustrated this:

*It started when he began messing up his medication, and through his doctor we contacted home-care services. And, you know, we could not manage without them. They are very gentle, nice and friendly, and my husband is so confident in them. But when it comes to a respite place, I wish they could try a little harder. When he says ‘no’, it is just accepted like that immediately, they say he can decide for himself since he is competent to provide consent. I wish they could try a little harder. But you know they are here for him, not for me*.

Others talked about the importance of being involved in making decisions. They considered it essential to be included in the planning and implementation of care; otherwise, they felt overruled, which lead to irritation and frustration. In addition, they emphasized the significance of getting to know the home-care personnel and needing to build trust and get attention themselves to talk about their situation. Lily said this around *involvement*:

*They entered our lives a decade ago to assist with showering. A while ago his condition deteriorated, and over my head they just decided for two carers to arrive here. I got mad then, so mad, because they had not asked me. I told them not to come here anymore and for a long time I did everything myself, but now they are back. It illustrates that I always wanted to be involved and decide. You know, many different carers visit, so it is difficult getting to know them, and they do not ask me how I currently experience the situation. I wish to have a reciprocal relationship of trust*.

The wives talked about a need for predictability; being able to plan and have a structure for the day were important. They wanted to organize and prepare themselves and the house before home-care personnel arrived. They preferred the house to look presentable and had elevated expectations for themselves. The housing arrangement affected how they could plan their daily living and influenced the complexity of their lives. Several of the older wives emphasized the inconvenience of just having one bathroom. Clara told this story about *predictability*:

*In recent years we have had to resort to using home-care services, and that is also a strain. You never know when they arrive, so you cannot plan. The house must be tidy, and everything must be ready. If I have had a bad night, I cannot sleep long because suddenly they are here. And you never know who will show up, there are so many different carers. I do not always know when I can do the morning routine myself because we have only one bathroom, and they take care of him there. Everything is unstable and uncertain and put on hold. What I wish for is more predictability*.

The stories conveyed how the home-care personnel did not pay attention to the wives during home visits, and how their need for predictability was not considered. The considerable number of different people visiting made it difficult to establish a relationship of trust between the wives and the home-care personnel. The wives wanted to have a primary home-care person to relate to, but unfortunately such relationships were rare.

## Discussion

The present findings demonstrate how the lives of the husbands and wives changed when their spouses became ill and how their narratives differed in what was experienced as being health-promoting. Differences in care style, coping style and experiences of caregiver burden are discussed below, along with the implications of the findings in the context of existing research as well as limitations of the study.

### Differences in care and coping styles

The present findings indicate how the husbands continued their everyday lives by learning new chores such as cooking and doing the laundry and domestic cleaning, in addition to performing the tasks that they previously had done. The importance of learning new tasks has been emphasized as a way to establish a new normality in old age [57]. This active and problem-solving approach to caring was described as task-oriented care and coping styles by Zygouri et al. [40], who found that by continuously solving challenges, older husbands ensured coherence in their everyday lives.

On the other hand, the wives in the current study generally considered it essential that they had time for themselves and sufficient social support to experience their everyday lives as comprehensible, manageable and meaningful. This need for time to themselves on their own terms can be understood as emotional and relational care and coping styles, since it involves an active way of identifying, processing and expressing one’s emotions [40].

The husbands considered that a managerial way of caring that promoted the stability of everyday life was valuable to them. Everyday life is understood as activities, habits and traditions [58]. This comprehension was further expanded by Almevall et al. [59] emphasizing how the home and everyday life are central to well-being. This seemed to apply to the older husbands in the present study, since the routines of everyday life were important to them. Bakken [60] described these minor everyday chores as life habits, while the philosopher de Beauvoir [61] argued that the importance of life habits increases with age, and can be enriching to give the world a certain quality and existential security. Life habits such as sharing a meal improved the relationship of the husbands in the present study with their wives by enhancing the feeling of togetherness. The appreciation older family caregivers have for their relationship with their spouses has been shown to be central to improving health [43].

Furthermore, the task-oriented caregiving approach can be understood as a way of gaining control in a changed situation. Robinson et al. [62] found that male caregivers positioned themselves by taking control and functioning as the head of the household, which in turn confirmed their experience of masculinity. In contrast, Andersen [63] found in a Norwegian report of older husbands’ experiences as caregivers that they were not concerned that they were men in the care situation, but rather emphasized the relationship with their wives and saw their position naturally based on how life had turned out. The husbands in the present study were concerned with and aware of traditional gender roles, such as by referring to a previous gender division of tasks. Their willingness to carry out both old and new tasks can perhaps be explained by the husbands being influenced by the shift in gender roles over recent decades, from a static male role to a more-fluid identity, which implies a more-dynamic understanding of the self [64]. By adapting to their changed lives, the husbands could maintain their identity as vigorous men who sorted things out, which helped to promote their health.

The wives had other experiences of health promotion. Their emotional way of caring and coping required personal time to allow them to adapt to the changed situation and regain mental balance on their own or through conversations with friends or family members, which ultimately promoted their health. This aligns with the WHO’s emphasis on independence, physical activity and social support as decisive determinants of health [65]. Jones et al. [66] also highlighted the importance of both male and female family caregivers having time for oneself and the opportunity to disconnect from caregiving responsibilities to experience peace of mind. Respite care was essential to the well-being of the older wives for addressing this issue. Our findings imply that the wives who had time for oneself and received practical support and respite seemed more satisfied than those who perceived these resources as inadequate. A very recent study similarly found that respite care reduced stress and helped to improve the relationship between older family caregivers and their spouses [67].

Based in Antonovsky’s theory of salutogenesis [46], the degree to which the care and coping styles of participants in the present study could promote health would depend on their SOC, and whether the general resistance resources are sufficient to meet their challenges. The older husbands described having coping resources to deal with their new everyday lives, and by maintaining and extending their responsibilities they promoted their own health. By having the capacity to narrate and reflect upon these everyday events as understandable, manageable and meaningful, this can be interpreted as a way to maintain a SOC, which can be described as crucial from a health-promoting perspective. In contrast, the older wives did not have sufficient time for themselves. They found it difficult to actively identify, process and express emotions, which probably threatened their SOC and their access to the resources required to cope with the challenges. Antonovsky [46] reported that having access to home-care personnel can be understood as a general resistance resource. Consequently, it is essential that these personnel take possible differences in care and coping styles into consideration when collaborating with family carers to provide health-promoting services.

### Differences in experiences of caregiver burden

The husbands’ stories about health promotion were framed in both the present and the past, highlighting good memories and demonstrating a more-positive attitude towards the changed situation compared to the wives. Conversely, the wives’ stories took place in the present and displayed how they perceived caring for their husband as a huge responsibility that resulted in them feeling physically and mentally exhausted in adapting to the needs of their husbands. They described having lost control over their freedom of movement and neglecting their own needs or interests, implying the presence of differences in the descriptions and experiences of caregiver burden. According to Choi and Seo [20], the concept of caregiver burden has several dimensions, and it seems like older wives experience much larger physical, emotional and social stresses. Our findings appear to align with other studies finding that wives report higher levels of caregiver burden [33, 37], depression and distress [38].

Differences in care and coping styles seemed to be related to the care burden experienced by the family caregivers in this study, which also affected their health-promoting experiences. Previous studies have indicated that the task-oriented and problem-solving coping style that is more typically exhibited by male caregivers can reduce stress [68]. Thus, those husbands in the present study who were skilled at performing tasks were less likely to experience stress and were more likely to experience improved health. The husbands pointed out there was a limit to the level of care that they could provide, but they had not reached that limit yet. In contrast, a prerequisite for the emotional and relational coping style of wives to diminish the experience of burden was personal space to regain balance by being alone or interacting with other people. According to Hong and Coogle [68], the wives adopting this coping style needed to be able to share their experiences in order to obtain emotional support and comfort.

The present findings also imply that the social support received by the older husbands and wives from the home-care personnel impacted the care burden they experienced and consequently their health. The husbands gave the impression of valuing this support, which seemed to reduce their experience of stress. Also, the wives valued the support that their husbands received, but at the same time reported that not being seen, heard and included by the home-care personnel increased their hardship. Several studies have found that older family caregivers sometimes experience exclusion by healthcare personnel [69, 70], and that inadequate social support can increase the care burden [35]. All of the participants in the present study considered adequate support from the home-care personnel to be crucial to their health, and research has illustrated that social support from healthcare personnel increases the experience of health promotion among family caregivers [45].

Previous studies have found that older husbands are often reluctant to ask for support and help from home-care services and their family, and are often unwilling to open up and talk about negative emotional dimensions of the care they provide [71], which might explain why the husbands in the present study reported that they received adequate support. On the other hand, one Norwegian study found that male caregivers received 19% more public assistance than did female caregivers [34]. This suggests the presence of gender discrimination, with the greater help being provided to men explaining why they experience a lower care burden. Allocating services based on gender would interfere with the fair distribution of the available care resources. However, the caregiving husbands in the present study did not appear to receive more informal or formal support than the caregiving wives; instead, they actually received fewer home-care visits per day despite all of the care-receivers in this study requiring extensive care.

The findings further demonstrated that the time for oneself available to the female caregivers was restricted by their husbands refusing respite care, which also increased the support required from home-care personnel. Resistance from the care-recipient is responsible for 38% of instances of respite care not being utilized [72]. In our study the home-care personnel generally accepted such resistance, which adversely affected the right to autonomy and fair treatment among older wives [73]. Families in Norway have a legislative right to publicly funded respite care if they meet specific criteria, such as providing long-term, extensive caregiving [18]. Since the care of family caregivers often is a precondition for older people to continue living at home, their need for and right to respite care should receive appropriate attention, especially since family caregivers are aging themselves and may have their own health challenges. These findings have thus uncovered a dilemma that needs the attention of home-care personnel to find appropriate solutions in individual cases.

The wives feeling of pressure to demonstrate proficiency in household chores when home-care personnel visited seemed to increase their experiences of a caregiver burden. These high self-expectations can be understood in the traditional view that women are generally expected to assume the caregiver role [32], and that the home is considered the woman’s domain [40, 68]. On the other hand, Pacheco et al. [41] found that while female caregivers do 60% more household chores than male caregivers, they reported the same satisfaction with their health and quality of life as men. However, Swinkels et al. [33] pointed out that the gender gap in the caregiver burden was not connected to the time spent on giving care, but instead was related to the difficulties that wives experience when combining different tasks such as caregiving and their own daily activities. Papastavrou et al. [25] highlighted that females expect more of themselves, and the healthcare services expect that women assume a caring role without training and receive less encouragement and praise, which can threaten the promotion of health. Conversely, another study [74] showed that older husbands put up boundaries when it comes to taking on responsibilities, because they believe that these belong to home-care personnel, which was also confirmed by several of the husbands in the present study. Based on our findings we would recommend that home-care personnel increase the attention and knowledge of these differences related to contributions and boundary setting to provide customized support and to strengthen the SOC and well-being of older family caregivers in their changed everyday lives.

### Limitations of the study

It was crucial to reach caregivers who provided extensive care, since they could offer nuanced and rich accounts of their experiences. To achieve a purposeful sample we collaborated with a municipal team in developing the inclusion criteria and recruiting participants. This team recruited the participants after a discussion with the four authors. Males and females from diverse socioeconomic backgrounds from different parts of the municipality were included. The inclusion criteria were developed to capture varied and detailed narratives from a diversity of caregiver experiences involving long-term, extensive responsibilities. The study might have overemphasized the hardships and underrepresented the experiences of caregivers who experienced fewer difficulties in their roles or who had positive experiences when performing their caregiving tasks.

The participants were interviewed only once, which potentially impaired the ability to establish trust and openness in speaking freely during the interviews. To mitigate this, the first author called each participant twice before the interview to provide them with information about the study, the researcher and the interview itself, and they were then asked if they had any relevant questions. Furthermore, the interviews were co-constructed between the participants and the first author, whose preconceptions could have influenced the interviews. The trustworthiness of the stories told by the first author was increased by describing preconceptions that might have influenced all parts of the study, such as those due to her background, knowledge of the field and reflections. The situations of the participants in the interviews were assessed in-depth by taking various approaches, such as asking open-ended questions, with the questions adapted based on the answers in order to promote a nuanced understanding. Several factors could have inhibited the participants from speaking openly. They might have felt pressured to present themselves in a positive light, leading them to exaggerate positive aspects of themselves to maintain a favourable self-image. Additionally, they might have feared that disclosing negative information about the care their spouses received from the home-care services could adversely affect subsequent care. This could also have been influenced by social desirability inducing participants to provide answers that they believed were socially acceptable or expected. This potential limitation was mitigated by ensuring confidentiality.

Trustworthiness was further promoted by including all authors in the interpretation and analysis of the stories. Detailed descriptions of the different stages of the research process were provided in order to promote transparency and thereby further strengthen the study [53]. Finally, the sample of the study was small, consisting of only ten participants. The goal was to gain in-depth insights into complex issues and understand the participants’ experiences and perspectives, rather than statistical generalize findings to a larger population. Therefore, we suggest that the study’s findings need to be interpreted with caution and viewed as providing preliminary insights into patterns rather than conclusive evidence. These patterns could be transferable to similar situations. The trustworthiness of the study was ensured by following the CASP (Critical Appraisal Skills Programme) checklist for qualitative studies [75].

## Conclusion

This study has provided insights into how ten older husbands and wives experienced health promotion as family caregivers to their spouses receiving home-care services. The findings suggest the presence of differences in care style, coping style, and in the experiences of the caregiver burden. The continuation of everyday life and social support were important for the husbands, while the wives considered that time for oneself and being seen, heard and included by the home-care personnel were essential to promoting their health. Several factors can influence these experiences, such as gender differences, cultural expectations influencing the provision of informal care, the relationship between the caregiver and the care-recipient, the health of the care-recipient and the health of the caregiver.

These findings imply that home-care services should customize their support by performing regular assessments of the different needs of older family caregivers. For husbands, interventions should include the extent of their practical workload and potential assistance, while for wives the attention should be focused on finding a balance between providing care and personal time. We further recommend that home-care personnel provide advice and support regarding the utilization of respite-care services. Improving the quality of support provided to older family caregivers at a system level and considering the caregivers’ experiences of health when providing such support will strengthen the health-promoting perspective and contribute to better health among older caregivers. This, in turn, can enable them to provide better care to their home-dwelling spouses.

Research targeting the development of systems at an organization level, such as the implementation of assessment tools that measure the needs of older family caregivers, is crucial to improving the quality of healthcare services. Due to the smallness of the sample, further qualitative and quantitative research on health-promoting experiences is needed to address the needs of older family caregivers while considering gender and other factors that can influence perspectives.

## Supplementary Information

Additional file 1: This file contains an extended version of the Interview guide.

## Data Availability

The transcripts and notes used and analysed during the current study are not publicly available to protect the anonymity of the participants and to maintain confidentiality. All data generated or analysed during this study are included in this published article. An extended version of the interview guide is available (Additional File 1).

## Abbreviations

ICF: International Classification of Functioning, Disability and Health
OECD: Organization of Economic Cooperation and Development
SOC: Sense of Coherence
WHO: World Health Organization
