# Supplementary material for: Narratives of health-promoting experiences by older husbands and wives providing care to their home-dwelling spouses receiving home-care services in Norway
Source: BMC Health Serv Res. 2024 Dec 18;24:1600. doi: 10.1186/s12913-024-12097-3 (PMC11653846; doi:10.1186/s12913-024-12097-3)
Supplement: Supplementary file 1 — Additional file 1: This file contains an extended version of the Interview guide. [file 12913_2024_12097_MOESM1_ESM.pdf]

Narratives of health-promoting experiences by older husbands and wives providing care to their home-dwelling spouses receiving home-care services in Norway

Interview guide

The purpose of this study is to understand the health-promoting experiences of older family caregivers who care for their home-dwelling spouses receiving home-care services. This project is a research project conducted by the University of South-Eastern Norway with external funding from the Research Council of Norway.

- Thank the participant for taking part in the study.
- Provide brief information about the first author, including name, qualifications, current position and relevant experience.
- Provide written and verbal information about the study. Retrieve informed written consent.
- Remind participants that their participation is voluntary, and that they can withdraw from the study at any time without any consequences.

Background information

Gender of family caregiver:

Gender of spouse/partner:

Age

Caregiver:

Spouse/partner:

Marital status

Married/partner:

Number of years in relationship:

Education

Family caregiver:

Spouse/partner:

Previous occupation

Family caregiver:

Spouse/partner:

Number of children:

Interview questions	
Please tell me how you have experienced being the wife/husband of … (name of husband/wife) after he/she became ill.<br><br>Additional subquestions:<br>• Can you tell me about an enjoyable moment you have experienced as a caregiver?<br>• Can you tell me about a difficult moment you have faced as a caregiver?<br>• How have your interactions with home-care services been?<br>• What could improve your current life situation?	
